# Stability and Accuracy Assessment of Identification of Traditional Chinese Materia Medica Using DNA Barcoding: A Case Study on Flos Lonicerae Japonicae

**DOI:** 10.1155/2013/549037

**Published:** 2013-06-05

**Authors:** Dianyun Hou, Jingyuan Song, Linchun Shi, Xiaochong Ma, Tianyi Xin, Jianping Han, Wei Xiao, Zhiying Sun, Ruiyang Cheng, Hui Yao

**Affiliations:** ^1^Institute of Medicinal Plant Development (IMPLAD), Chinese Academy of Medical Sciences & Peking Union Medical College, No. 151 Malianwa North Road, Haidian District, Beijing 100193, China; ^2^Agricultural College, Henan University of Science and Technology, Luoyang, Henan 471003, China; ^3^Jiangsu Kanion Pharmaceutical Co. LTD, Lianyungang, Jiangsu 222001, China; ^4^State Key Laboratory of New-Tech for Chinese Medicine Pharmaceutical Process, Lianyungang 222001, China; ^5^College of Chinese Medicine, Shandong University of Traditional Chinese Medicine, Jinan 250355, China; ^6^Beijing University of Chinese Medicine, Beijing 100102, China

## Abstract

DNA barcoding is a novel molecular identification method that aids in identifying traditional Chinese materia medica using traditional identification techniques. However, further study is needed to assess the stability and accuracy of DNA barcoding. Flos Lonicerae Japonicae, a typical medicinal flower, is widely used in China, Korea, and other Southeast Asian countries. However, Flos Lonicerae Japonicae and its closely related species have been misused and traded at varying for a wide range of prices. Therefore, Flos Lonicerae Japonicae must be accurately identified. In this study, the ITS2 and *psbA-trnH* regions were amplified by polymerase chain reaction (PCR). Sequence assembly was performed using CodonCode Aligner V 3.5.4. The intra- versus inter-specific variations were assessed using six metrics and “barcoding gaps.” Species identification was conducted using BLAST1 and neighbor-joining (NJ) trees. Results reveal that ITS2 and *psbA-trnH* exhibited an average intraspecific divergence of 0.001 and 0, respectively, as well as an average inter-specific divergence of 0.0331 and 0.0161. The identification efficiency of ITS2 and *psbA-trnH* evaluated using BLAST1 was 100%. Flos Lonicerae Japonicae was formed into one clade through the NJ trees. Therefore, Flos Lonicerae Japonicae can be stably and accurately identified through the ITS2 and *psbA-trnH* regions, respectively.

## 1. Introduction 

*Lonicera japonica *Thunb. (Caprifoliaceae) is a commonly used herb that grows widely in China, Japan, Korea, and Southeast Asian countries [1]. The flower bud, called Flos Lonicerae Japonicae, is the most important part of the plant for medicinal use [2, 3], with more than 3000 years of medical history. Flos Lonicerae Japonicae was first reported in one of the earliest pharmacopoeias worldwide, Shen-Nong's Herbals Classic [4]. Flos Lonicerae Japonicae is considered a herbal medicine with heat-clearing, detoxifying, and anti-inflammatory effects [5, 6]. In addition, extracts of Flos Lonicerae Japonicae have shown efficacy against various diseases such as H1N1 influenza [4, 7–9], hand-foot-and-mouth disease (HFMD) [4, 10], and severe acute respiratory syndromes, among others [11–13]. In recent years, Flos Lonicerae Japonicae has also become the main material of a Chinese “cooling” beverage [14]. Flos Lonicerae, which comes from *L. macranthoides *Hand-Mazz, *L. fulvotomentosa* Hsu et S.C. Cheng,* L. confusa* (Sweet) DC., and *L. hypoglauca* Miq., is another medicinal material from *Lonicera *[6]. More than 30% of the current traditional Chinese medicine prescriptions have recently used Flos Lonicerae Japonicae [4]. Consequently, Flos Lonicerae and other species of *Lonicera*, such as *L. japonica* var. *chinensis*, *L. similis*, and *L. acuminata*, have been misused as Flos Lonicerae Japonicae because of high market demand for Flos Lonicerae Japonicae.

High-quality botanical materials are important for the safe and effective use of herbal products [15]. Several criteria and techniques have been used in the identification of Flos Lonicerae Japonicae to ensure its quality and therapeutic effects [16–19]. Li et al. introduced the morphologic and microstructural methods for identifying *L. japonica* and *Lonicerae *[20]. Despite its ability to preliminarily identify *L. japonica,* the method needs the integrity of the material structure. The geoherbalism of *L. japonica* has been identified by sequence divergence of the 5S-rRNA gene spacer region [21]. However, Flos Lonicerae Japonicae is difficult to identify according to morphology, microstructural methods, and certain molecular markers.

DNA barcoding can quickly and reliably identify species using a standard genetic sequence that can be amplified by universal primers from a small fragment of the genome [22–25]. Several DNA regions have been evaluated for potential application as DNA barcodes, such as *matK*, *rbcL*, *psbA-trnH*, and ITS2 [26–28]. In the recent years, the DNA barcoding technology has been increasingly used in several fields including species identification [29–33], biodiversity study [34, 35], misidentification detection [36], food safety testing [14], and tackling illegal trade of endangered species [37, 38]. Chen et al. suggested that ITS2 can be used as a novel DNA barcode for the identification of medicinal plants and their closely related species [27]. The potential identification ability of ITS2 in plants has been assessed by analyzing 50,790 plant ITS2 sequences [28]. The China Plant BOL Group has also shown that ITS2 can be used to identify seed plants [39]. ITS2 sequences are potential general phylogenetic markers and have been widely used for phylogenetic reconstructions at both the genus and the species levels [40, 41]. Previous studies used the *psbA-trnH* intergenic spacer region from plastid DNA to successfully resolve a number of species identification cases [42–45]. Chen et al. recommended *psbA-trnH* as a complementary barcode to ITS2 for a broad series of plant taxa [27].

In our previous study, the genomic DNA extracted from the leaves of *L. japonica *was used for genomic identification. Sun et al. tested several potential DNA regions and proposed that *psbA-trnH* can be used for the identification of *L. japonica *and its closely related species [46]. In the present study, the ITS2 and *psbA-trnH* barcoding regions of Flos Lonicerae Japonicae and closely related species were evaluated for the suitability for species discrimination.

## 2. Materials and Methods

### 2.1. Materials

Flower bud samples from 45 individuals belonging to seven species, which were used as Flos Lonicerae Japonicae and Flos Lonicerae in Chinese markets, were collected from various geographical areas in China, including Henan, Shandong, and Guizhou (Table 1). All samples were identified by Professor Yulin Lin from the Institute of Medicinal Plant Development (IMPLAD), Chinese Academy of Medical Sciences. Voucher specimens were deposited in the IMPLAD herbarium in Beijing, China. Subsequently, eight ITS2 sequences of *L. japonica* were downloaded from GenBank for further analysis.

### 2.2. DNA Extraction, PCR Amplification, and Sequencing

Total genomic DNA was extracted from 25 mg of dry flower bud by the Plant Genomic DNA Kit (Tiangen Biotech Co., Beijing, China) after grinding for 1 min at 30 oscillations/s using a DNA Extraction Grinder (MM 400; Retsch, Haan, Germany). PCR amplifications of the ITS2 region were performed in a Peltier Thermal Cycler PTC0200 (BioRad Lab Inc., USA) using a pair of primer ITS2F (5′-ATGCGATACTTGGTGTGAAT-3′) and ITS3R (5′-GACGCTTCTCCAGACTACAAT-3′) [27]. PCR reactions were performed in a 25 *μ*L volume containing approximately 20–50 ng of genomic DNA, 2× EasyTaq PCR SuperMix (TransGen Biotech Co., China), 1.0 *μ*L of each primer (2.5 *μ*M), and distilled deionized water. The reaction conditions were as follows: 95°C for 5 min, 40 cycles at 94°C for 30 s, 58°C for 30 s, 72°C for 45 s, and 72°C for 10 min. The *psbA-trnH* was conducted as described previously [27, 42]. The desired PCR products were separated by electrophoresis in 1.2% agarose gels. The purified PCR products were directly sequenced bidirectionally by the sequencing center at Molecular Biology and Chemical (Mbchem, Shanghai, China).

### 2.3. Data Analysis

The forward and reverse trace files were trimmed and assembled after sequencing using the CodonCode Aligner V 3.5.4 (CodonCode Co., USA). The ITS2 region was annotated with the HMMer software using ITS2 database [47]. The genetic distances were then calculated using MEGA 5.0 [48], according to the Kimura 2-Parameter (K2P) model. The average intra-specific distance, theta, and coalescent depth were computed to evaluate the intraspecific variation by the K2P model [49]. The average interspecific distance, minimum interspecific distance, and theta primer were used to represent interspecific divergences [24, 27]. The distribution of intra- versus inter-specific variability was assessed using the DNA barcoding gaps [50]. Species identification efficiency was determined based on BLAST1 method [51]. NJ trees were constructed to identify Flos Lonicerae Japonicae and its closely related species. 

## 3. Results 

### 3.1. Efficiency of PCR Amplification

**Figure 1:**
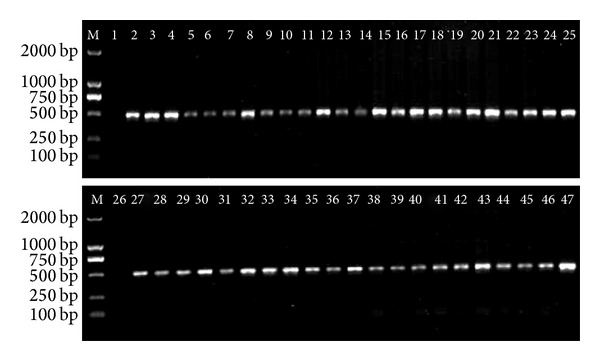
PCR amplification results of the ITS2 regions of *L. japonica *and its related species. M: marker; 1 and 26: negative control (CK); 2–25: *L. japonica*; 27–31: *L. macranthoides*; 32–36: *L. fulvotomentosa*; 37-38: *L. hypoglauca*; 39-40: *L. confuse*; 41–45: *L. similis*; 46-47: *L. acuminate. *

DNA was isolated from 45 individuals. Some smear bands can only be found by DNA gel electrophoresis because of DNA degradation. The PCR amplification success rates for ITS2 and *psbA-trnH* were both 100% (Figure 1). The sequencing results showed that high-quality bidirectional trace files were obtained from the ITS2 regions.

### 3.2. Analysis of DNA Divergence

The ITS2 sequence lengths of 24 Flos Lonicerae Japonicae samples were 228 bp, and the average GC content was 75.4%. Only one nucleotide variation site was found at the 27th base, which is the substitution of C/T. Two new haplotypes of ITS2 sequences generated in this study were submitted to GenBank, with the following accession numbers: JX669516 and JX669517. The ITS2 regions of haplotype A1 were the same as that of JX669516, whereas that of haplotype A2 was the same as that of JX669517. According to the K2P model, the intraspecific distance of Flos Lonicerae Japonicae ITS2 ranged from 0 to 0.0044. The ITS2 sequence length of Flos Lonicerae and other samples ranged from 228 bp to 229 bp; the average GC content was 73.9%. A total of 14 nucleotide variation sites and one insertion/deletion mutation were detected in 53 ITS2 sequences (Table 2). In this study, the ITS2 region of the other *Lonicera* species except *L. japonica* can be divided into six haplotypes (Table 1). These ITS2 regions were also submitted to GenBank, and GenBank accession numbers were obtained (Table 1).

Using the same approach, also* psbA-trnH* sequences of the 45 *Lonicera* samples were analyzed (Table 2). The sequence length of the *psbA-trnH* of Flos Lonicerae Japonicae was 339 bp, and the average GC content was 29.5%. No variation sites were found in the *psbA-trnH* regions. Additional characteristics of the *psbA-trnH* sequences of 45 samples are shown in Table 2.

Ideal barcode sequences should provide a distinct inter-specific divergence and relatively small intra-specific variation. They must concurrently show significant differences between these two types of variation to distinguish individual intra-specific variation from variation between species. According to the K2P model, the genetic distance of Flos Lonicerae Japonicae from its closely related species was evaluated using the ITS2 regions. The inter-specific distance was 0.0331, which is larger than the intra-specific distance of 0.0010. The minimum inter-specific distance was 0.0142, which is larger than coalescent depth of 0.0019 (Table 3). Likewise, the minimum inter-specific distance for the *psbA-trnH* was 0.0060, which is larger than coalescent depth of 0.0004 (Table 3).

### 3.3. Assessment of Barcoding Gap

**Figure 2:**
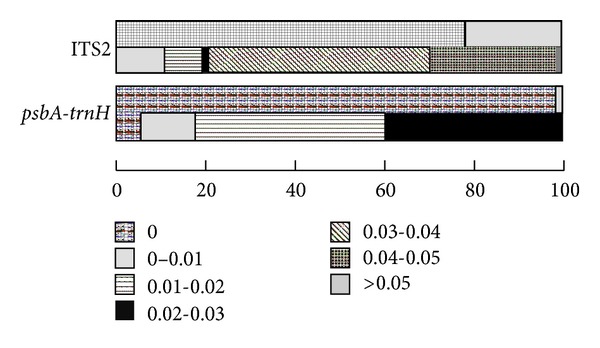
Relative distribution of inter-specific divergence between Flos Lonicerae Japonicae and intra-specific variation for ITS2 and *psbA-trnH *sequences. The two bars in the box represent intra-specific (above) and inter-specific (below) genetic distances.

We investigated the distribution of genetic distance in classes of 0.010 distance units for ITS2 and *psbA-trnH* sequences to perform a preliminary examination of intra- versus inter-specific variation. The results showed apparent gaps between the intra- and inter-specific variability of ITS2. Obvious gaps were also found between the intra- and inter-specific variability of *psbA-trnH *using the same method (Figure 2).

### 3.4. Applicability for Species Discrimination

**Figure 3:**
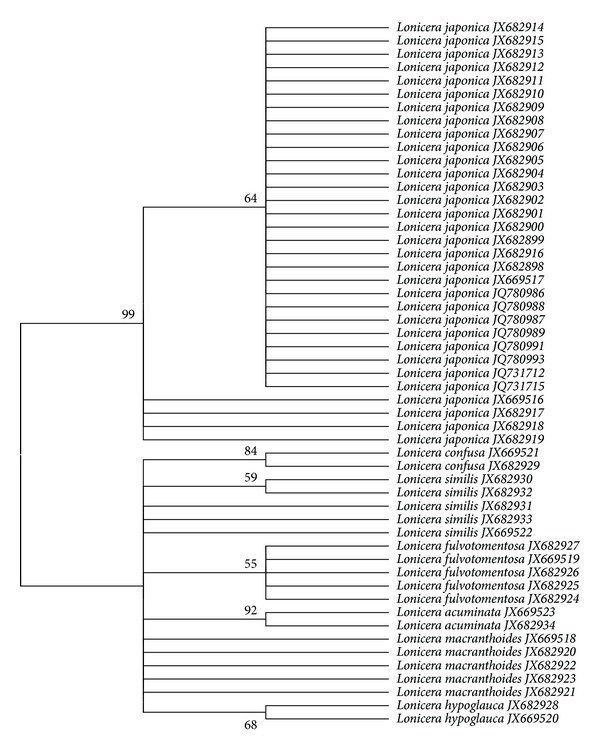
Phylogenetic tree of Flos Lonicerae Japonicae and its closely related species constructed with the ITS2 sequences using NJ method. The bootstrap scores (1 000 replicates) are shown (≥50%) for each branch.

Two methods of BLAST1 and NJ tree techniques were used to evaluate the reliability of species identification using DNA barcoding. The results indicated that the ITS2 and *psbA-trnH* regions exhibited the highest identification efficiency (100%) by BLAST1 (Table 3). NJ tree effectively determines the power of a given locus combination to discriminate among species. In this study, a phylogenetic tree was constructed using the NJ method, with 1000 bootstrap replicates for the ITS2 and *psbA-trnH* sequences (Figure 3, Supplementary Figure  1 available online at: http://dx.doi.org/10.1155/2013/549037). The results showed that *L. japonica* formed into one clade, which can be distinguished successfully from its closely related species.

## 4. Discussion 

An ideal barcoding system should have sufficient variation among the sequences to discriminate species; however, it also needs to be sufficiently conserved to achieve less variability within species than between species [52]. In addition, the DNA barcode should be easily amplified using one pair of universal primers; identification should be rapid, accurate, cost effective, and easy to use even for a nonspecialist [53]. The applicability of the ITS2 regions for species identification has been tested by Chen et al. [27]. In recent years, the DNA barcoding technique has become the main method for species identification [54–56].

In the present study, the ITS2 region was used for the first time to identify Flos Lonicerae Japonicae and its closely related species. The samples were collected from the main producing areas of the species. All samples were obtained from the flower bud, which is the medicinal part of *L. japonica*. The following results were obtained from this study. First, the length of the ITS2 of Flos Lonicerae Japonicae was 228 bp, which is short and relatively easy to amplify using one pair of universal primers. Second, the ITS2 regions of Flos Lonicerae Japonicae obtained from different areas were similar; only one variable site was found. The minimum inter-specific distance was 0.0142, which is significantly larger than coalescent depth of 0.0019. Divergence at the inter-specific level and relatively low divergence at the intra-specific level (Table 3) were found. Third, the identification efficiency of the 53 samples was 100% by the BLAST1 method. The results obtained using the NJ tree can also be used to distinguish Flos Lonicerae Japonicae successfully. In summary, the ITS2 sequence can be used as a DNA barcode for identifying Flos Lonicerae Japonicae and its closely related species. To date, ITS2 regions have been widely used to distinguish related species. The study by Gao et al. [31] indicated that ITS2 regions can be used as an efficient and powerful marker for distinguishing various species in Fabaceae. Pang et al. accurately identified the Ephedrae herb and its closely related species by the ITS2 DNA barcoding technique [57]. The stability and accuracy of identifying Notopterygii Rhizoma et Radix has been tested using the ITS/ITS2 barcodes [55]. All results consistently showed that the ITS2 DNA barcode exhibits good stability and accuracy in identifying Chinese materia medica.

A previous study by Sun et al. used the leaves of *L. japonica* and proposed the *psbA-trnH* regions for the identification of *L. japonica* and its related species [46]. We obtained genomic DNA from the flowers of Flos Lonicerae Japonicae and assessed their PCR amplification and identification efficiency to test the *psbA-trnH* regions. The results revealed that *psbA-trnH* can also be used to identify Flos Lonicerae Japonicae and verified the results of Sun et al. [46]. This study simultaneously showed that ITS2 regions can be used to distinguish Flos Lonicerae Japonicae. However, in the study by Sun et al., the resulting PCR product of ITS2 was not amplified using the approach employed by Chen et al. [27]. Compared with the study by Sun et al., the present study obtained two findings. One is that genomic DNA, which can be improved for follow-up experiments, was extracted by extending the pyrolysis time. The other is that the ITS2 regions were amplified successfully by increasing the annealing temperature from 56°C to 58°C using a universal primer and a 2× EasyTaq PCR SuperMix (TransGen Biotech Co., China). Sequence analysis indicated that the average GC contents of Flos Lonicerae Japonicae was 75.4%, which may be that reason Sun et al. failed to obtain the ITS2 regions by the Chen et al. method [27].

Several studies have reported the existence of multiple copies of ITS2 in plants and animals [58]. Questions have been raised whether the sequence obtained by PCR would be stable and representative as a DNA marker. Alvarez and Wendel suggested that the presence of different copies of ITS2 can result in misleading phylogenetic inferences [58]. However, Coleman indicated that ITS2 can be effectively treated as a single locus [59]. The most recent study also revealed that using the major variants alone is sufficient for phylogeny construction and species determination in most cases although intragenomic multiple variants are frequently found within each genome. Inclusion of minor variants further improves the resolution of species identification [60]. In the present study, the PCR amplification success rates for ITS2 was 100% and high-quality sequence stability from the 45 samples was obtained. This result indicates that the major variants alone are sufficient for PCR amplification of the ITS2 region. Therefore, the ITS2 DNA barcode can be used to identify Flos Lonicerae Japonicae and its closely related species.

The results of this study can help identify Flos Lonicerae Japonicae and Flos Lonicerae in the market and ensure safe and stable supply of traditional medicines derived from *L. japonica*. These findings can be used as reference for the authentication of other medicinal herbs, especially the flower types. The results can also be valuable in resource protection, industrial production, customs, forensic examination, and plant quarantine.

## 5. Conclusions

ITS2 and *psbA*-*trnH* were examined for their stability and accuracy in identifying Chinese materia medica in the case study with Flos Lonicerae Japonicae. The results indicate that using the ITS2 and *psbA-trnH* regions as DNA barcodes can stably and accurately distinguish Chinese materia medica as well as provide a new technique to ensure clinical safety in using traditional Chinese medicines.

## Supplementary Material

Flos Lonicerae Japonicae is a traditional Chinese materia medica which has an important function in the treatment of H1N1 influenza, hand-foot-and-mouth disease (HFMD), and severe acute respiratory syndromes (SARS). However, Flos Lonicerae Japonicae and its closely related species have been misused. Therefore, it is imperative to identify them accurately. DNA barcoding is a novel molecular identification method that provides a turning point in solving the difficulty in identifying traditional Chinese materia medica using traditional identification techniques. However, the stability and accuracy of DNA barcoding need further study. The present study aims to examine the stability and accuracy of the ITS2 and *psbA-trnH* regions in the identification of Flos Lonicerae Japonicae and its closely related species. The results showed that both regions can stably and accurately distinguish Flos Lonicerae Japonicae and its closely related species.

## Figures and Tables

**Table 1:** Samples used in the present study.

Latin name	Haplotype (for ITS2)	Voucher no.	GenBank no. ITS2, *psbA-trnH *	Locality
*L. japonica *	A1	YC0014MT15	JX669516, JX885527	Pingyi, Shandong
*L. japonica *	A1	YC0014MT60	JX682917, JX885540	Pingyi, Shandong
*L. japonica *	A1	YC0014MT91	JX682918, JX885545	Kaixian, Chongqing
*L. japonica *	A1	YC0014MT104	JX682919, JX885525	Huanggang, Hubei
*L. japonica *	A2	YC0014MT16	JX669517, JX885528	Pingyi, Shandong
*L. japonica *	A2	YC0014MT29	JX682898, JX885529	Xixia, Henan
*L. japonica *	A2	YC0014MT31	JX682899, JX885530	Xixia, Henan
*L. japonica *	A2	YC0014MT32	JX682900, JX885531	Xixia, Henan
*L. japonica *	A2	YC0014MT33	JX682901, JX885532	Xixia, Henan
*L. japonica *	A2	YC0014MT41	JX682902, JX885533	Fengqiu, Henan
*L. japonica *	A2	YC0014MT43	JX682903, JX885534	Fengqiu, Henan
*L. japonica *	A2	YC0014MT54	JX682904, JX885535	Donghai, Jiangsu
*L. japonica *	A2	YC0014MT55	JX682905, JX885536	Donghai, Jiangsu
*L. japonica *	A2	YC0014MT57	JX682906, JX885537	Donghai, Jiangsu
*L. japonica *	A2	YC0014MT58	JX682907, JX885538	Donghai, Jiangsu
*L. japonica *	A2	YC0014MT59	JX682908, JX885539	Donghai, Jiangsu
*L. japonica *	A2	YC0014MT62	JX682909, JX885541	Pingyi, Shandong
*L. japonica *	A2	YC0014MT63	JX682910, JX885542	Pingyi, Shandong
*L. japonica *	A2	YC0014MT64	JX682911, JX885543	Nanning, Guangxi
*L. japonica *	A2	YC0014MT81	JX682912, JX885544	Liupanshui, Guizhou
*L. japonica *	A2	YC0014MT92	JX682913, JX885546	Wanzhou, Chongqing
*L. japonica *	A2	YC0014MT98	JX682914, JX885547	Zhoukou, Henan
*L. japonica *	A2	YC0014MT101	JX682915, JX885524	Shucheng, Anhui
*L. japonica *	A2	YC0014MT108	JX682916, JX885526	Julu, Hebei
*L. japonica *	A2	—	JQ731712	GenBank
*L. japonica *	A2	—	JQ731715	GenBank
*L. japonica *	A2	—	JQ780993	GenBank
*L. japonica *	A2	—	JQ780991	GenBank
*L. japonica *	A2	—	JQ780986	GenBank
*L. japonica *	A2	—	JQ780987	GenBank
*L. japonica *	A2	—	JQ780988	GenBank
*L. japonica *	A2	—	JQ780989	GenBank
*L. macranthoides *	B1	YC0163MT10	JX669518, JX885548	Liuzhi, Guizhou
*L. macranthoides *	B1	YC0163MT20	JX682920, JX885549	Banan, Chongqing
*L. macranthoides *	B1	YC0163MT21	JX682921, JX885550	Longhui, Hunan
*L. macranthoides *	B1	YC0163MT25	JX682922, JX885551	Nanjiang, Sichuan
*L. macranthoides *	B1	YC0163MT26	JX682923, JX885552	Puer, Yunan
*L. fulvotomentosa *	B2	YC0164MT01	JX669519, JX885553	Anlong, Guizhou
*L. fulvotomentosa *	B2	YC0164MT07	JX682924, JX885554	Xingren, GuiZhou
*L. fulvotomentosa *	B2	YC0164MT08	JX682925, JX885555	Zhenfeng, GuiZhou
*L. fulvotomentosa *	B2	YC0164MT09	JX682926, JX885556	zerong, GuiZhou
*L. fulvotomentosa *	B2	YC0164MT11	JX682927, JX885557	Xingyi, GuiZhou
*L. hypoglauca *	B3	YC0165MT01	JX669520, JX885558	Nanning, Guangxi
*L. hypoglauca *	B3	YC0165MT02	JX682928, JX885559	Hezhou, Guangxi
*L. confusa *	B4	YC0166MT04	JX669521, JX885560	Shipan, Sichuan
*L. confusa *	B4	YC0166MT07	JX682929, JX885561	Jianyang, Sichuan
*L. similis *	B5	YC0169MT04	JX669522, JX885562	Chengkou, Chongqing
*L. similis *	B5	YC0169MT07	JX682930, JX885563	Kaixian, Chongqing
*L. similis *	B5	YC0169MT10	JX682931, JX885564	Chengkou, Chongqing
*L. similis *	B5	YC0169MT11	JX682932, JX885565	Chengkou, Chongqing
*L. similis *	B5	YC0169MT16	JX682933, JX885566	Kaixian, Chongqing
*L. acuminata *	B6	YC0170MT01	JX669523, JX885567	Jianyang, Sichuan
*L. acuminata *	B6	YC0170MT04	JX682934, JX885568	Guangyuan, Sichuan

**Table 2:** Sequence characteristics of ITS2 and *psbA-trnH* of Flos Lonicerae Japonicae and its related species.

Sequence characteristics	ITS2	*psbA-trnH *
Length range in Flos Lonicerae Japonicae (bp)	228	339
Length in all taxa (bp)	228-229	332–356
Average of GC content in Flos Lonicerae Japonicae (%)	75.4	29.5
Average of GC content in all taxa (%)	73.9	29.6
No. of variable sites in Flos Lonicerae Japonicae	1	0
No. of variable sites in all taxa	14	10

**Table 3:** Inter- and intra-specific genetic divergence and identification efficiency in ITS2 and *psbA-trnH* sequences of *L. japonica *and its related species.

Parameter	ITS2	*psbA-trnH *
All inter-specific distance	0.0331 ± 0.0119	0.0161 ± 0.0066
Theta primer	0.0253 ± 0.0093	0.0126 ± 0.0050
The minimum inter-specific distance	0.0142 ± 0.0105	0.0060 ± 0.0060
All intra-specific distance	0.0010 ± 0.0018	0.0000 ± 0.0003
Theta	0.0011 ± 0.0093	0.0002 ± 0.0004
Coalescent depth	0.0019 ± 0.0024	0.0004 ± 0.0011
Identification efficiency (BLAST1)	100%	100%
