# Supplementary material for: Stability and Accuracy Assessment of Identification of Traditional Chinese Materia Medica Using DNA Barcoding: A Case Study on Flos Lonicerae Japonicae
Source: Biomed Res Int. 2013 Jun 5;2013:549037. doi: 10.1155/2013/549037 (PMC3687729; doi:10.1155/2013/549037)
Supplement: Supplementary file 1 — Flos Lonicerae Japonicae is a traditional Chinese materia medica which has an important function in the treatment of H1N1 influenza, hand-foot-and-mouth disease (HFMD), and severe acute respiratory syndromes (SARS). However, Flos Lonicerae Japonicae and its closely related species have been misused. Therefore, it is imperative to identify them accurately. DNA barcoding is a novel molecular identification method that provides a turning point in solving the difficulty in identifying traditional Chinese materia medica using traditional identification techniques. However, the stability and accuracy of DNA barcoding need further study. The present study aims to examine the stability and accuracy of the ITS2 and psbA-trnH regions in the identification of Flos Lonicerae Japonicae and its closely related species. The results showed that both regions can stably and accurately distinguish Flos Lonicerae Japonicae and its closely related species. [file 549037.f1.doc]

Fig.S1. Phylogenetic tree of Flos Lonicerae Japonica and its closely related species constructed with the *psbA*-*trnH* regions using NJ method. The bootstrap scores (1 000 replicates) are shown (≥50%) for each branch.
